# Treatment of Pruritus Vulvæ

**Published:** 1872-02

**Authors:** 


Treatment of Pruritus Vulvæ.—Those who have had any experience in the treatment of this troublesome affection will learn with interest that Mr. McGrath states (Canada Lancet, November, 1871,) that he has found the following, applied by means of a soft sponge after ablution, morning and evening, attended with the most satisfactory and speedy result: Biborate of soda, ʒ ij; hydrochlorate of morphia, gr. xx; hydrocyanic acid, ʒ j; glycerine, f ℥ j; distilled rose-water, f ℥ viij.

